# Integrated transcriptomics contrasts fatty acid metabolism with hypoxia response in β-cell subpopulations associated with glycemic control

**DOI:** 10.1186/s12864-023-09232-5

**Published:** 2023-03-28

**Authors:** Mario A Miranda, Juan F Macias-Velasco, Heather Schmidt, Heather A Lawson

**Affiliations:** Department of Genetics, Washington University School of Medicine, 660 South Euclid Ave, Campus Box 8232, Saint Louis, MO 63110 USA

**Keywords:** Single-cell RNAseq, Bulk RNAseq, Hyperglycemia, Insulin, β-cell heterogeneity, Diabetes, Obesity, Mouse model

## Abstract

**Background:**

Understanding how heterogeneous β-cell function impacts diabetes is imperative for therapy development. Standard single-cell RNA sequencing analysis illuminates some factors driving heterogeneity, but new strategies are required to enhance information capture.

**Results:**

We integrate pancreatic islet single-cell and bulk RNA sequencing data to identify β-cell subpopulations based on gene expression and characterize genetic networks associated with β-cell function in obese SM/J mice. We identify β-cell subpopulations associated with basal insulin secretion, hypoxia response, cell polarity, and stress response. Network analysis associates fatty acid metabolism and basal insulin secretion with hyperglycemic-obesity, while expression of *Pdyn* and hypoxia response is associated with normoglycemic-obesity.

**Conclusions:**

By integrating single-cell and bulk islet transcriptomes, our study explores β-cell heterogeneity and identifies novel subpopulations and genetic pathways associated with β-cell function in obesity.

**Supplementary Information:**

The online version contains supplementary material available at 10.1186/s12864-023-09232-5.

## Introduction

Proper insulin secretion from pancreatic β-cells is required to maintain glycemic control. Obesity initially promotes β-cell expansion, but prolonged glycemic stress and inflammation drive β-cell death and dysfunction, resulting in type 2 diabetes [1–3]. Without sufficient β-cell mass and insulin production, sustained hyperglycemia increases risk for deadly metabolic diseases [4–6]. Currently, transplantation of cadaveric islets is the only method for restoring β-cell mass in diabetes but is severely limited by donor availability and requires lifelong immunosuppressant therapy [7, 8]. Differentiating induced pluripotent stem cells into insulin secreting cells may alleviate this bottleneck, but current methods fail to recapitulate fine-tuned glucose sensing and insulin secretion in vivo [9, 10]. There is urgent need for therapies that improve endogenous β-cell function. This requires understanding how and why β-cells become dysfunctional in obesity.

Improving endogenous β-cell function in diabetes is complicated by cellular heterogeneity, because individual β-cells vary significantly in function, gene expression, protein level, and stress response [11]. Single cell technologies permit interrogation of cellular heterogeneity, including identification of functionally distinct β-cell subpopulations and characterization of response to obesity. Several research groups have proposed subpopulations based on clustering using single cell RNA sequencing (scRNA-seq), however, there is little agreement among studies [12–15]. High rates of gene dropout and low read depth contribute to these problems, necessitating approaches that improve information capture without losing cell type-specific information [16, 17].

Integrating sc-RNAseq with bulk RNAseq data leverages bulk sequencing’s high read depth, allowing for capture of lowly expressed genes and robust expression analysis. This is particularly important for bulk RNAseq from isolated islets, where variable levels of exocrine tissue contamination can confound differential expression analysis. Several tools can integrate sc- and bulk RNA-seq data, focusing on deconvoluting bulk RNAseq data from heterogeneous tissue to account for differences in tissue composition [12, 18, 19]. These methods estimate and control for cell type abundance but do not identify cell type-specific expression signatures in bulk datasets. Analytical strategies that identify cellular gene expression signatures in bulk RNAseq data allow for robust cell type-specific differential expression analysis and complex network analysis that is currently not feasible in scRNA-seq data.

Here, we characterize gene expression in β-cells from obese SM/J mice, who spontaneously transition from hyperglycemic to normoglycemic with improved β-cell function between 20 and 30 weeks of age [20, 21]. We assign functional identities to 4 subpopulations of β-cells using scRNA-seq. These subpopulations vary in proportion among hyperglycemic-obese (20-week high fat-fed), normoglycemic-obese (30-week high fat-fed), and normoglycemic-lean mice (20-week low fat-fed). We identify 316 genes specifically expressed by β-cells and establish a β-cell gene expression profile for each mouse cohort. We leverage this information to focus our analyses of bulk-islet RNAseq from a larger sample size of mice that were deeply phenotyped for obesity and glycemic traits as well as β-cell function. We identify β-cell-specific differential expression and gene networks associated with glycemic state in the hyperglycemic-obese and normoglycemic-obese mice. A novel potential regulator of β-cell function, *Pdyn* (Prodynorphin), is primarily expressed by a β-cell subpopulation associated with normoglycemic-obesity, while a genetic network associated with fatty acid metabolism is overexpressed in hyperglycemic-obesity. This analysis demonstrates that integrating scRNAseq with bulk RNAseq is a powerful approach for exploring β-cell heterogeneity and identifying key genes and subpopulations that strongly associate with glycemic state. The genetic networks and β-cell subpopulation signatures we identify have high potential to lead to further research aimed at improving β-cell function in obesity.

## Results

### SM/J islets contain four β-cell subpopulations

**Fig. 1:**
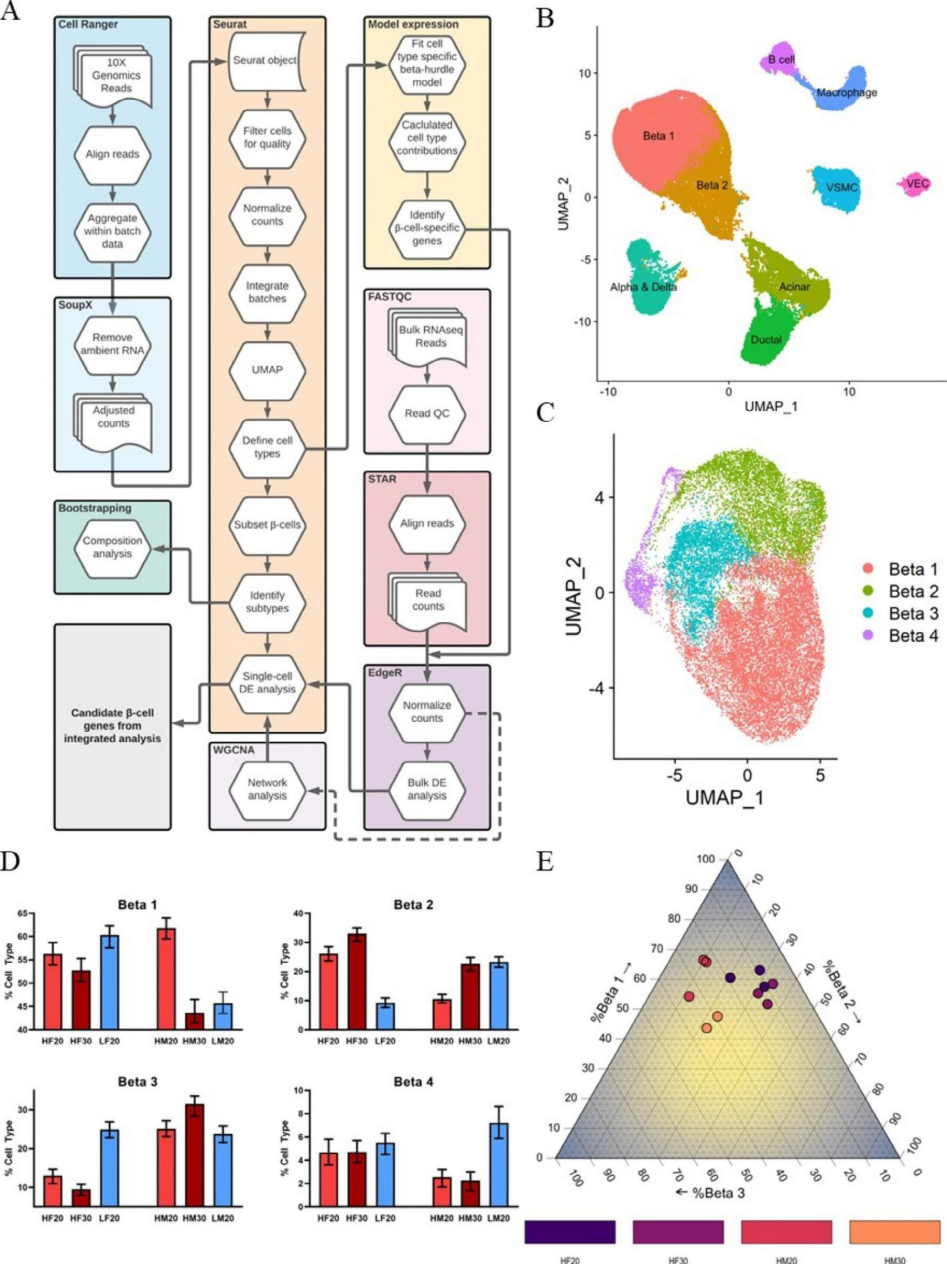
SM/J islets contain four subpopulations of β-cells. **(A)** Data analysis pipeline identifies subpopulations of β-cells and integrates bulk and single cell RNA sequencing data. **(B)** Single cell RNA sequencing UMAP plot of islet tissue. VSMC – vascular smooth muscle cell, VEC – vascular endothelial cell. **(C)** Single cell RNA sequencing UMAP of 4 subpopulations of β-cells. **(D)** Bootstrap analysis quantifies relative proportions of β-cell subpopulations across cohorts. Bar plot represents actual proportion, error bars illustrate 95% confidence interval. Cell type composition estimates with non-overlapping confidence intervals are significantly different. **(E)** Ternary plot illustrates subtype composition of Beta 1–3 in high-fat SM/J mice. HF20–20wk high-fat female, HF30–30wk high-fat female, HM20–20wk high-fat male, HM30–30wk high-fat male, LF20–20wk low-fat female, LM20–20wk low-fat male

We developed an analysis pipeline that integrates sc- and bulk RNA-seq data to characterize the transcriptional changes in β-cells from obese SM/J mice as they improve glycemic control with age (Fig. 1A). We identified islet cells (α, β, and δ), exocrine cells (acinar and ductal), vascular cells (smooth muscle and endothelial), and immune cells (B cells and macrophages) (Fig. 1B) using expression of known marker genes (**Supplemental Figure**S1F). Subsequent clustering of β-cells revealed 4 subpopulations, labeled Beta 1–4 (Fig. 1C, **Supplemental Figure**S1G). 20wk hyperglycemic high-fat obese males have a significantly larger Beta 1 population compared to 30wk normoglycemic high-fat obese males and 20wk low-fat lean males, at the expense of a diminished Beta 2 population (Fig. 1D-E).

### β-cell subpopulations have unique expression signatures

**Fig. 2:**
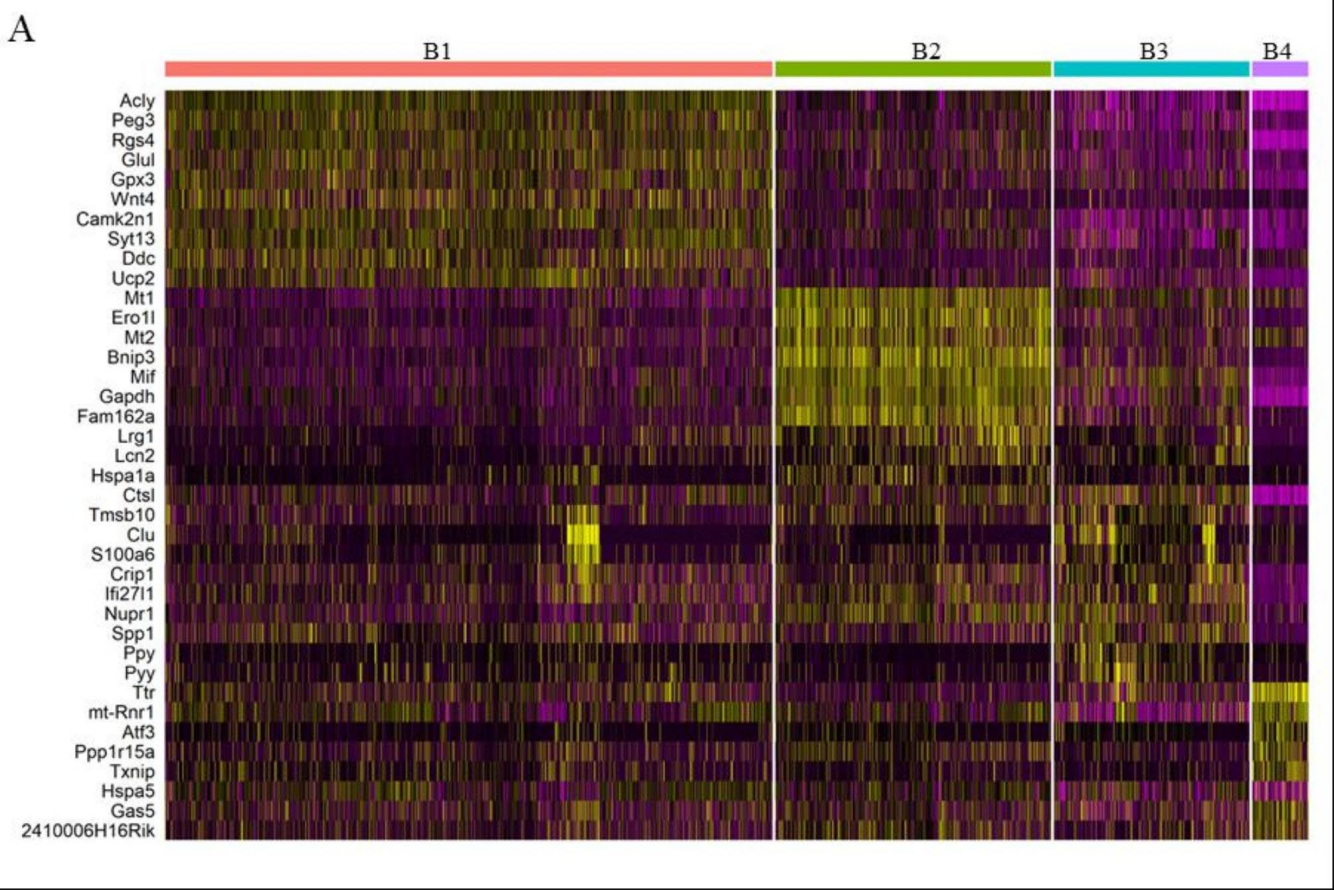
β-cell subpopulations have unique gene expression signatures. **(A)** Heat map for top 10 differentially expressed genes in each β-cell subpopulation (B1-4). Yellow is highly expressed; purple is lowly expressed

To identify genes that are overexpressed in each subpopulation, we performed differential expression analysis between each subpopulation and all other β-cells (**Supplemental Table**S1). The top ten highest differentially expressed genes within each subtype are visualized in Fig. 2A. Gene enrichment analysis on the overexpressed genes in each subpopulation reveals potential specialization: Beta 1 – basal insulin secretion, Beta 2 – hypoxia response, Beta 3 – cell polarity, Beta 4 – stress response (Table 1).

**Table 1:** Top five results for significantly enriched genes within each β-cell subpopulation, including gene ontology terms

Gene Set	Description	Ratio	FDR
Beta 1			
GO:0023061	signal release	4.60	6.89E-13
GO:0009914	hormone transport	4.72	3.23E-09
GO:0030133	transport vesicle	3.90	3.45E-07
GO:0019932	s-messenger-mediated signaling	4.34	3.45E-07
GO:0033500	carbohydrate homeostasis	3.89	4.43E-05
Beta 2			
GO:0070482	response to oxygen levels	5.49	7.22E-08
GO:0006090	pyruvate metabolic process	10.55	6.13E-07
GO:0046939	nucleotide phosphorylation	10.45	2.33E-06
GO:0009132	nucleoside diphosphate metabolic process	8.84	2.55E-06
GO:0006091	generation of precursor metabolites and energy	4.53	3.34E-06
Beta 3			
GO:0016327	apicolateral plasma membrane	31.35	0.004258
GO:0003735	structural constituent of ribosome	7.90	0.004258
GO:0005840	ribosome	5.42	0.02957
GO:0044445	cytosolic part	5.42	0.02957
GO:0031012	extracellular matrix	4.66	0.02957
Beta 4			
GO:0043620	regulation of DNA-templated transcription in response to stress	37.35	0.000216
GO:0045926	negative regulation of growth	12.11	0.004441
GO:0035966	response to topologically incorrect protein	16.41	0.004441
GO:0042594	response to starvation	11.63	0.017622
GO:1,901,654	response to ketone	11.33	0.094767

### SM/J β-cells uniquely express 316 genes

**Fig. 3:**
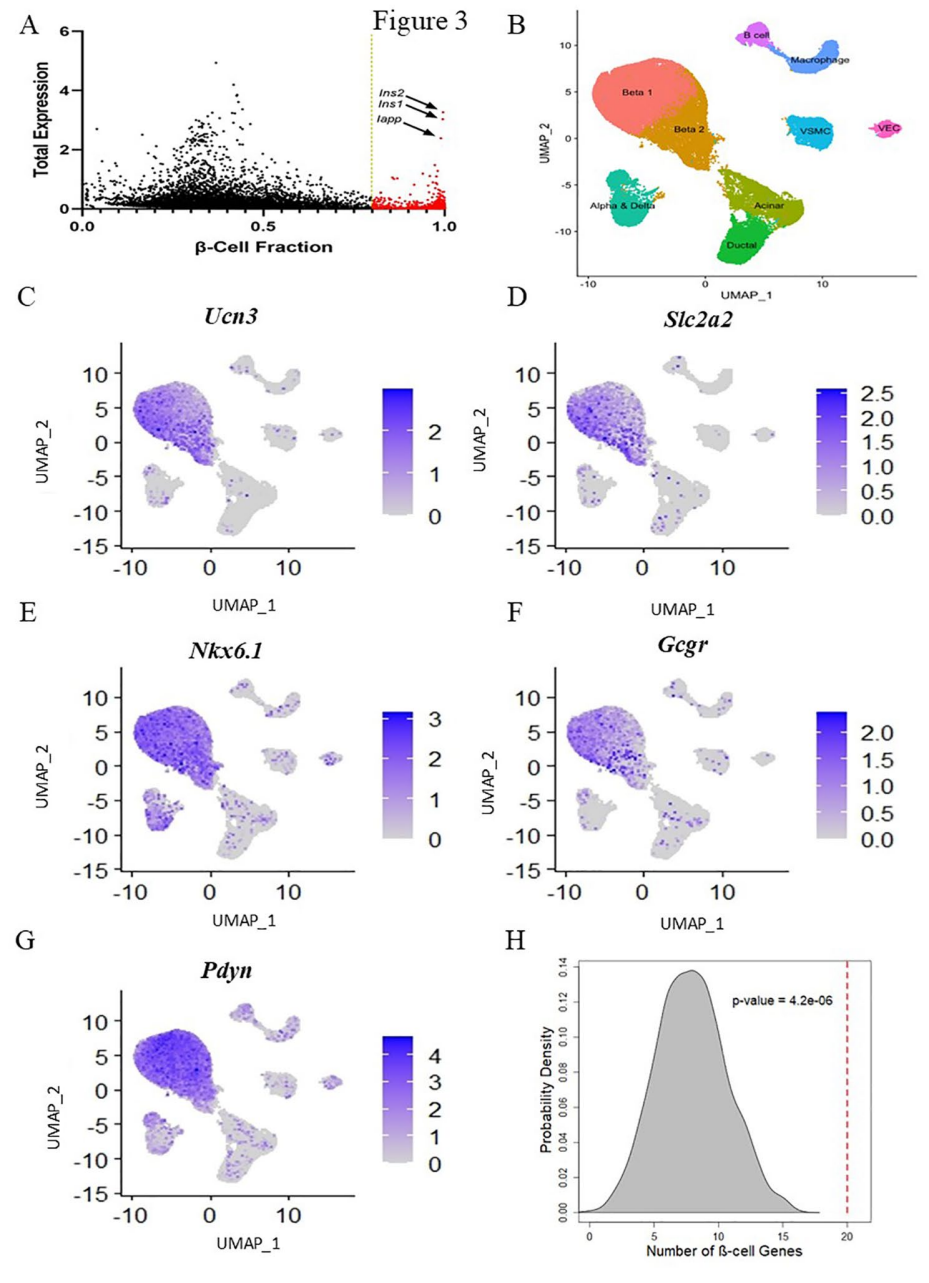
Single cell RNA sequencing identifies β-cell-specific genes. **(A)** Total gene expression and β-cell-specific contribution to gene expression in 20wk low-fat female β-cells. Gold line indicates threshold for β-cell-specific expression. Red dots identify genes that pass the threshold cutoff in this cohort. Arrows identify highly expressed genes associated with β-cell identity. Genes must pass threshold in all cohorts to be considered β-cell-specific for further analysis. **(B)** UMAP plot of all islet cell types. UMAP plots for β-cell-specific expression of known β-cell markers **(C)***Ucn3*, **(D)***Slc2a2*, **(E)***Nkx6.1*, and **(F**) *Gcgr*. UMAP plot for β-cell-specific expression of novel β-cell marker **(G)***Pdyn*. **(H)** Permutation analysis of β-cell-specific genes in Beta 1 overexpressed genes. Distribution shows expected number of β-cell-specific genes in Beta 1 overexpressed gene set based on chance (n = 1000 permutations), red line indicates real number of β-cell-specific genes in Beta 1 overexpressed gene set. P-value indicates probability of number of β-cell-specific genes in Beta 1 overexpressed gene set due to chance

To identify genes primarily expressed by β-cells in scRNA-seq data, we employed a beta hurdle model, which allowed us to estimate the relative contribution of each cell type to total gene expression (**Supplemental Figure**S2). To be considered a β-cell-specific gene, we required β-cells to account for at least 80% of total expression within each cohort (Fig. 3A, see Identification of β-cell-specific genes in methods). We identified 316 β-cell-specific genes (**Supplemental Table**S5), comprised of genes canonically associated with β-cell identity, including *Ucn3*, *Slc2a2*, *Nkx6.1*, and *Gcgr* (Fig. 3B-F) and genes with unknown function in β-cells including *Pdyn* (Fig. 3G). Overrepresentation analysis suggests enrichment for terms associated with β-cell function, including mature onset diabetes of the young (MODY) and carbohydrate homeostasis, along with terms related to neuron function including neuroactive ligand-receptor interaction, neuron projection terminus, and axon part (Table 2). We then sought to discover if β-cell-specific genes were overexpressed within any of the β-cell subpopulations. We identified 20 β-cell-specific genes overexpressed in Beta 1 cells, far more than would be expected by chance (Fig. 3H).

**Table 2:** Top five results for over-representation analysis (ORA) for β-cell-specific genes, including gene ontology and mammalian phenotype ontology terms

Gene Set	Description	Ratio	FDR
mmu04950	Maturity onset diabetes of the young	8.38	0.0808
mmu04080	Neuroactive ligand-receptor interaction	5.05	0.0808
GO:0044306	neuron projection terminus	3.97	0.0808
GO:0033500	carbohydrate homeostasis	3.19	0.0808
GO:0033267	axon part	2.48	0.0808

### β-cell genes are differentially expressed by age, diet, and sex

**Fig. 4:**
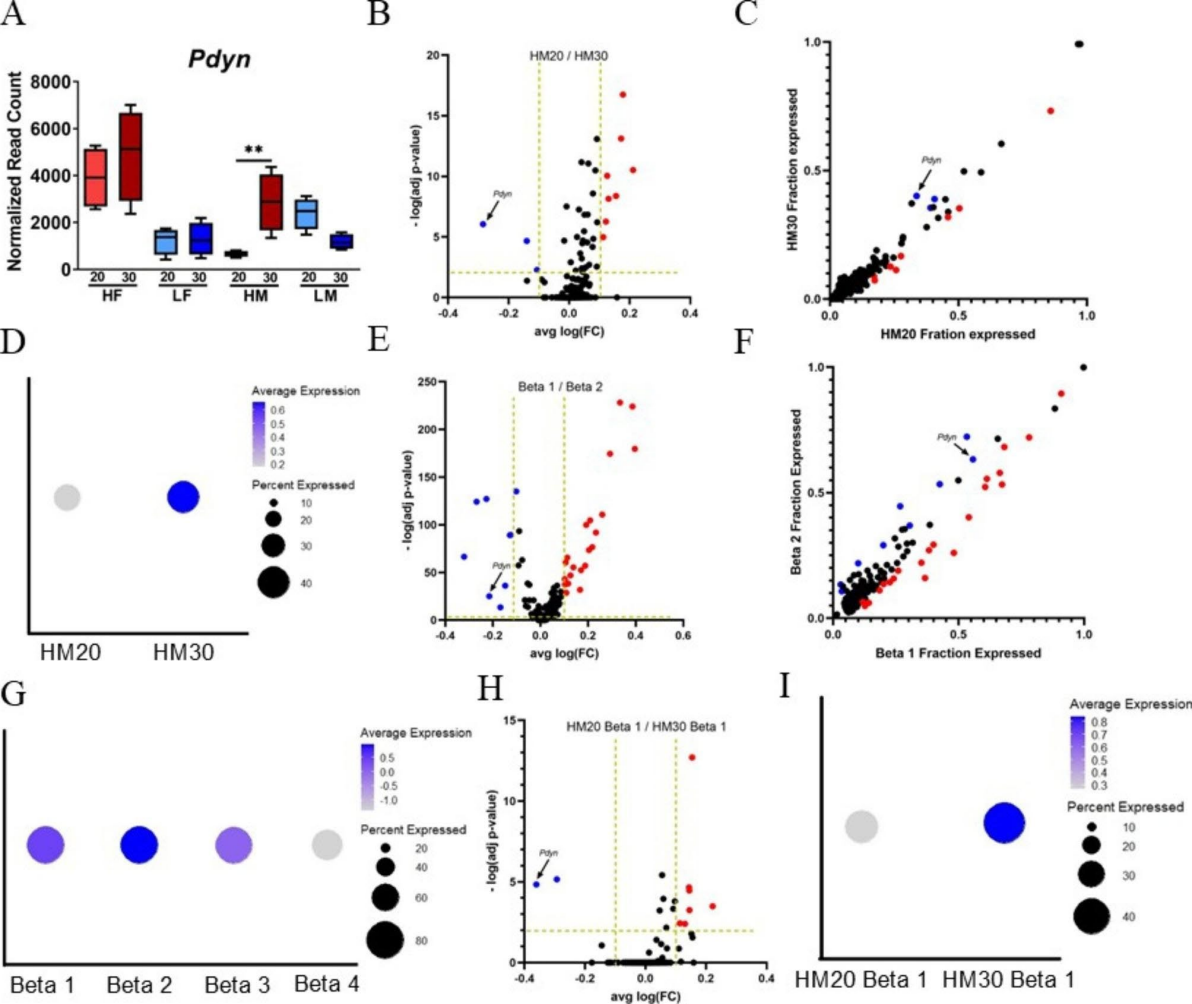
*Pdyn* is differentially expressed between and within β-cell subtypes. **(A)***Pdyn* is differentially expressed in high-fat males in bulk RNAseq data. **(B)** Differential expression of β-cell-specific genes across all β-cells between 20wk and 30wk high-fat males in single cell RNAseq data. **(C)** Percent of β-cells in 20wk and 30wk high-fat males expressing β-cell-specific genes. **(D)** Total expression of *Pdyn* across all β-cells in 20- and 30wk high-fat males. **(E)** Expression of β-cell-specific genes between Beta 1 and Beta 2 cells across all individuals. **(F)** Percent of Beta 1 and Beta 2 cells expressing β-cell-specific genes across all individuals. **(G)** Total *Pdyn* expression in Beta 1 and Beta 2 cells across all individuals. **(H)** Expression of β-cell-specific genes in Beta 1 cells between 20wk and 30wk high-fat males. **(I)** Total *Pdyn* expression in Beta 1 cells across in 20wk and 30wk high-fat males. **p-value < 0.01 in FDR corrected pairwise comparison. HF20–20wk high-fat female, HF30–30wk high-fat female, HM20–20wk high-fat male, HM30–30wk high-fat male, LF20–20wk low-fat female, LM20–20wk low-fat male, LF30–30wk low-fat female, LM30–30wk low-fat male. Blue genes are significantly under-expressed in comparison, red genes are significantly over-expressed. Vertical golden lines indicate threshold for significance based on average log fold change, horizontal line indicates threshold for significance based on Bonferroni-corrected p-value

To robustly characterize β-cell gene expression in SM/J mice, we normalized bulk RNA-seq data from 20- and 30-week high- and low-fat males and females (4 per cohort, n = 32) using only the 316 β-cell-specific genes. Principal components analysis on the normalized expression data revealed high-fat males separated from other cohorts (**Supplemental Figure**S3A). This is consistent with our previous studies showing that high-fat fed SM/J males show a more extreme glycemic response than other cohorts [20, 21]. Pairwise comparison revealed 8 differentially expressed genes between 20- and 30wk high-fat males and 2 differentially expressed genes between 20- and 30wk high-fat females (**Supplemental Figure**S3B). 20wk male mice differentially express 104 genes between diets, while 30wk male mice differentially expressed 17. Females differentially expressed a largely consistent set of genes between diets in 20- and 30wk cohorts. Differential expression analysis is reported in **Supplemental Table**S6. We focused subsequent analyses between 20wk and 30wk high-fat males (**Supplemental Figure**S3C), comparing hyper- and normoglycemic-obese mice. We highlight a gene with unknown roles in β-cell function, *Pdyn*, as differentially expressed between 20- and 30wk high-fat males (Fig. 4A).

### Pdyn is differentially expressed between and within β-cell subpopulations

We sought to determine if the differential expression of *Pdyn* seen in the bulk analysis is recapitulated at the single cell level. We compared expression of *Pdyn* in all β-cells between 20wk high-fat males and 30wk high-fat males (Fig. 4B, **Supplemental Table**S2) and found significant differential expression, including differences in the proportion of β-cells expressing *Pdyn* (Fig. 4C), visualized in Fig. 4D. To determine if differential expression could be attributed to differences in *Pdyn* expression between β-cell subpopulations, we calculated differential expression between the two most prominent subtypes, Beta 1 and Beta 2, revealing significant differential expression of *Pdyn* (Fig. 4E, **Supplemental Table**S3) including differences in the proportion of β-cells expressing *Pdyn* (Fig. 4F), visualized in Fig. 4G. This suggests differential expression of *Pdyn* can be attributed to differences in subpopulation proportions. To determine if differential expression could be attributed to differences in *Pdyn* expression within β-cell subpopulations, we calculated differential expression within Beta 1 and Beta 2 cells between 20wk and 30wk high-fat males, revealing significant differential expression of *Pdyn* in Beta 1 cells (Fig. 4H, **Supplemental Table**S4), visualized in Fig. 4I. *Pdyn* is not differentially expressed in Beta 2 cells between 20wk and 30wk high-fat males (data not shown). These findings suggest differential expression of *Pdyn* is driven by differences in expression between subpopulations and within subpopulations during the resolution of hyperglycemia in obese male mice.

### β-cell gene expression networks correlate with metabolic traits

We performed network analysis to characterize how groups of genes behaved across age and dietary cohorts. We performed weighted gene co-expression network analysis (WGCNA), which groups similarly co-expressed genes into discreet modules, then correlates these modules with phenotypes [22]. We collected metabolic phenotypes in the bulk RNA-seq mice including body weight, blood glucose level, serum insulin, and islet-specific phenotypes including glucose-stimulated insulin secretion, basal insulin secretion, and islet insulin content (**Supplemental Figure**S4A-F). Performing co-expression analysis identified 9 discreet modules of genes, 6 of which correlated significantly with at least one phenotype (**Supplemental Table**S7, **Supplemental Figure**S5). We highlight the blue module for its correlation with blood glucose levels.

### Blue module associated with fatty acid metabolism correlates with blood glucose level and is altered by age in obese mice

**Fig. 5:**
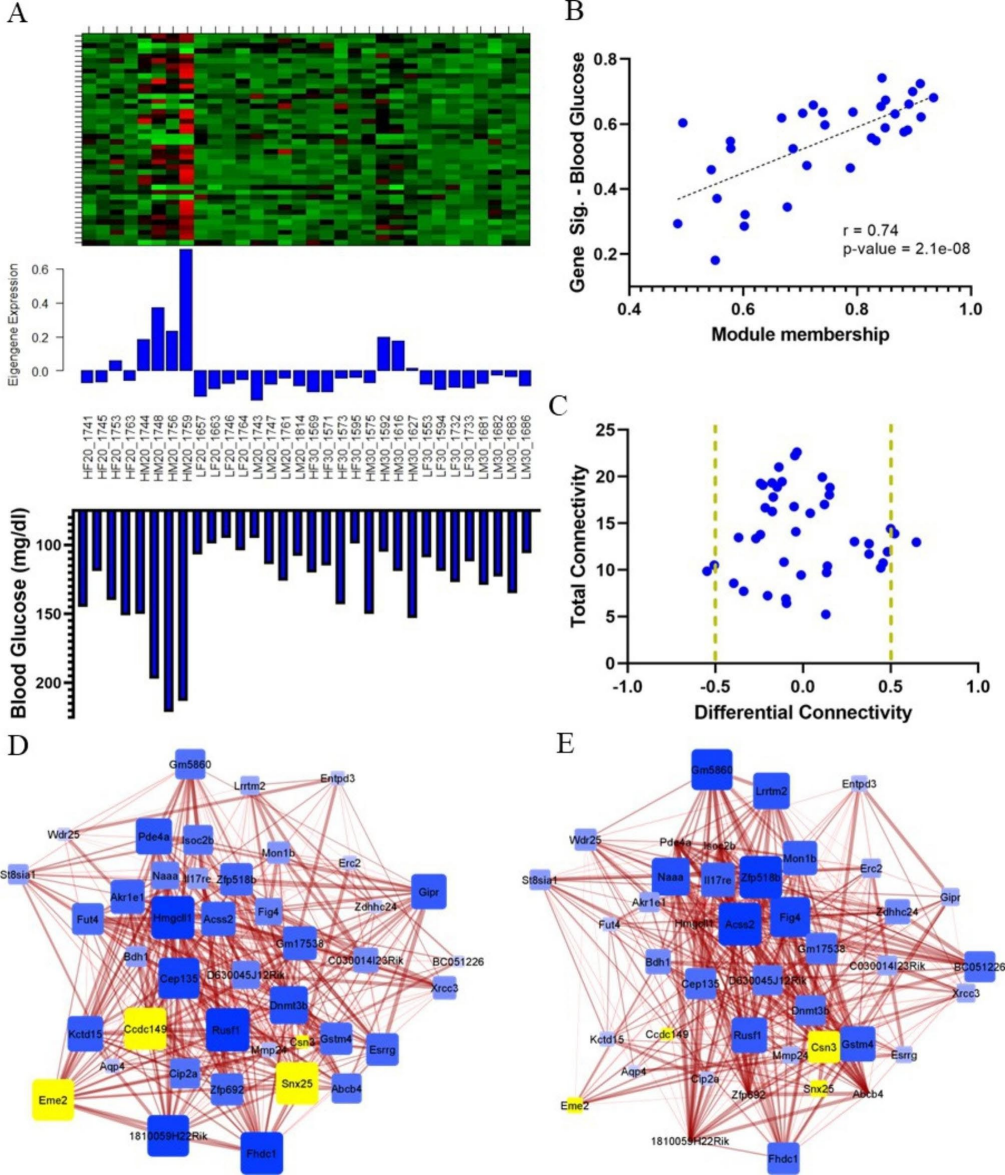
Blue modules network structure is altered by age in high-fat male mice. **(A)** Blue module heatmap, eigengene expression, and blood glucose levels across all individuals. **(B)** Correlation between strength of module membership and blood glucose levels for blue module. **(C)** Total and differential connectivity between blue module genes in 20wk and 30wk high-fat male mice. Vertical golden lines indicate threshold for differential connectivity. **(D)** Blue module network structure in 20wk high-fat males and **(E)** 30wk high-fat males. Size and color of node indicates overall connectivity within the network, thickness of edges indicates strength of correlation between gene pairs. Differentially connected genes highlighted in yellow

The blue module contains 42 genes, and over-representation analysis reveals suggestive enrichment for genes related to fatty acid metabolism (Table 3). Blue network eigengene expression correlates with blood glucose level across all mice (Fig. 5A, **Supplemental Figure**S6A). This correlation is driven by the resolution of hyperglycemia in high-fat mice at 30 weeks of age (Fig. 5A, **Supplemental Figure**S6B). Strength of individual gene membership within the blue module correlates with strength of correlation to blood glucose levels (Fig. 5B). The blue network is visualized as the sum of gene-pair correlations (**Supplemental Figure**S6C). Four genes were identified as differentially connected between networks in 20wk high-fat hyperglucemic obese males and 30wk high-fat normoglycemic obese males, although none were individually differentially expressed between 20- and 30wk high-fat males (Fig. 5C, **Supplemental Table**S8). Subset networks are visualized for 20wk high-fat males (Fig. 5D) and 30wk high-fat males (Fig. 5E), with differentially connected genes highlighted in yellow.

**Table 3:** Top five results for over-representation analysis (ORA) for blue module genes, including gene ontology and mammalian phenotype ontology terms

Gene Set	Description	Ratio	FDR
mmu00072	Synthesis and degradation of ketone bodies	72.437	0.096371
mmu00601	Glycosphingolipid biosynthesis	39.005	0.1765
mmu00650	Butanoate metabolism	31.691	0.17977
mmu04976	Bile secretion	14.913	0.60456
mmu01100	Metabolic pathways	2.1102	1

## Discussion

Pancreatic β-cell heterogeneity has been studied extensively using single cell technology because of the cell type’s unique insulin-secreting capabilities and central role in diabetes etiology. Several groups have proposed the existence of functionally distinct β-cell subpopulations [23–26], but their existence and relevance to insulin homeostasis remain controversial. We identified 4 β-cell populations (Figs. 1 and 2, **Supplemental Figure**S1), Beta 1–4, across hyperglycemic-obese, normoglycemic-obese, and normoglycemic-lean mice. While the functional roles assigned to each population are based on gene enrichment analysis and open to interpretation (Table 1), the relative proportion of each population changes in conjunction with islet function across cohorts, suggesting a functional relevance to overall β-cell population structure.

Beta 1 cells are most prevalent in hyperglycemic obese mice and are associated with elevated basal insulin secretion and low insulin content. Beta 1 cells overexpress mature markers including *Ucn3* [27], *Pdx1* [28], and *Acly* [29], and negative regulators of glucose-stimulated insulin secretion including *Abcc8* [30], *G6pc2* [31], and *Ucp2* [32]. Terms associated with Beta 1 cells include signal release and hormone transport. Beta 1 cells appear to be primed for insulin release, but not to perform glucose-stimulated insulin secretion. Farack et al. [24] identified a subpopulation of “extreme” β-cells that specialize in basal insulin secretion, with high insulin expression and low mature insulin content. These cells have high expression of markers *Ucn3*, *Acly*, and *Pdx1*, and increase in proportion in obese mice. The Beta 1 cells we identified are similar to “extreme” β-cells based on their mature profile, potentially limited glucose response, and abundance in diabetic obese mice. Future studies will determine if these β-cells function by over-expressing insulin secretion pathway components while suppressing glucose response mechanisms.

Beta 2 cells are prevalent in normoglycemic-obese and normoglycemic-lean mice, and are associated with high GSIS and high islet insulin content. Beta 2 cells are enriched for response to oxygen levels, pyruvate metabolism, and nucleotide phosphorylation, each associated with protection from hypoxia [33, 34]. These cells are equally prevalent in obese 30wk high-fat males and lean 20wk low-fat males, which differ greatly in islet mass, suggesting Beta 2 cells are not a hypoxic population. Further, obese 20wk high-fat males have similar islet mass to obese 30wk high-fat males, but a much smaller Beta 2 population. Importantly, 30wk high-fat males have much healthier glycemic parameters than 20wk high-fat males [20, 21]. Several groups have identified a subset of heavily vascularized islets that have elevated oxygen consumption and superior GSIS at the cost of susceptibility to hypoxia. We suspect Beta 2 cells represent a highly functional β-cell population that confer protection against hypoxia [35, 36]. Given their abundance in functional islets from both obese and lean normoglycemic mice, we hypothesize Beta 2 cells represent a mature, and possibly stress-resistant β-cell population.

Beta 3 cells are prevalent in normoglycemic obese mice which have elevated β-cell mass, high GSIS, and high insulin content. Beta 3 cells overexpress plasma membrane components, including several claudin family members, and ribosome components. Claudins provide structural integrity to tight junctions, which maintain cell polarity [37]. Polarity in β-cells serves as both a driver and a characteristic of mature, highly functional β-cells [23, 38, 39]. *Cldn4, Cldn3*, and *Cldn7* are all upregulated in Beta 3 cells and associated with mature β-cell function [40]. Beta 3 cells upregulate 7 ribosome genes and elevated ribosome biogenesis is associated with increased β-cell apoptosis [41], β-cell replication [23], and mature β-cell function [42], making the functional relevance of overexpression unclear. One gene, *Rpl7*, is associated with mature β-cell function [43], while *Rpl23* protects against apoptosis [44]. From this we conclude that Beta 3 cells represent a polarized and mature β-cell population.

Beta 4 cells have the lowest abundance and are elevated in lean males compared to obese males. Terms associated with Beta 4 cells include response to stress and response to topologically incorrect protein. In addition to overexpression of UPR components, Beta 4 cells have significant down regulation of *Ins1* and *Ucn3*. Several groups have identified β-cell subpopulations based on a stress response signature [13, 45, 46], which have coalesced into a theory about stress response cycling, where β-cells go through periods of UPR activation and low insulin production to clear misfolded proteins [15, 47]. Interestingly, 20wk low-fat mice have a similar proportion of stress response cycling cells compared to other groups (~ 7%), however this population is significantly smaller in high-fat males, suggesting that stress response cycling may be suppressed by obesity.

Efforts to describe β-cell transcriptional heterogeneity are marred by lack of consensus across scRNA-seq studies [48]. This is attributed to low read depth, resulting in differences in gene capture driven by technical artifacts and random chance rather than true biological variation [16]. Further, it is unclear what level of gene expression fold change is meaningful between individual cells, rendering single cell data poorly suited to assess differential expression across multiple cohorts. To address these problems, we developed a technique to integrate single cell data with bulk RNA-seq data (Fig. 3, **Supplemental Figure**S2), which is not inhibited by these technical limitations, to assess β-cell-specific differential expression across cohorts of high- and low-fat fed male and female SM/J mice. While the list of genes identified as β-cell-specific is only 316 genes (**Supplemental Table**S5), we are highly confident of their specificity to β-cells, allowing for robust assessment of differential expression, network analysis, and most importantly, association with metabolic variation and function. Sub-setting the bulk RNA-seq data by β-cell-specific genes provided a small but highly focused search space empowered to identify genes that influence β-cell function in hyperglycemic-obesity, normoglycemic-obesity, and healthy lean mice.

We identified *Pdyn* as a novel candidate gene associated with improved glycemic control in males (Fig. 4). *Pdyn* encodes Prodynorphin, which is cleaved into dynorphin A and dynorphin B, and exerts effects through the κ-opioid receptor [49]. Mice deficient in *Pdyn* have enhanced obesity and hyperglycemia when placed on a high fat diet, without altering feeding behavior, suggesting dynorphins modulate metabolism in obesity [50]. While *Pdyn*’s role in β-cell function is unknown, activation of the κ-opioid receptor reduces hyperglycemia in diabetic mice and β-cells secrete dynorphin A in a glucose-dependent manner [51, 52]. Further, *Pdyn* expression is associated with regulation by *Pax6* and *Lkb1*, hallmarks of mature β-cells [53, 54]. *Pdyn* is over expressed in Beta 2 cells, which are increased in normoglycemic-obese mice, and may link improved β-cell function with hypoxia response. *Pdyn* expression provides protection from hypoxia in lung and neuronal tissue, and dynorphins increase oxygen availability through vasodilation, which could enhance aerobic respiration [55–57].

We explored if subpopulation composition contributes to differential expression of *Pdyn* (Fig. 4). Total *Pdyn* expression increased across all β-cells between 20 and 30wk high-fat fed males, in agreement with the bulk RNA-seq data. *Pdyn* is overexpressed in Beta 2 cells, which increase in proportion between 20 and 30 weeks in high-fat mice, suggesting differential expression of *Pdyn* is driven by a change in subpopulation proportions. Further, Beta 1 cells increase expression of *Pdyn* between 20 and 30 weeks, suggesting they are becoming more Beta 2-like, and potentially improving in function. These findings underscore the complexity of gene expression and suggest it is important to consider both subpopulation composition and expression within subpopulations when exploring β-cell heterogeneity in diabetes and obesity.

We used weighted gene co-expression network analysis (WGCNA) to identify networks of co-expressed β-cell genes and to assess how networks correlate with metabolic and islet phenotypes (**Supplemental Figure**S4). Previous efforts to construct networks in islet RNA-seq data failed to account for cell-type specific gene expression, making it difficult to determine if these networks operate within an individual cell [58–60]. Our analysis revealed 6 β-cell-specific modules that correlate with phenotypes, one of which is enriched for intriguing ontology terms (**Supplemental Figure**S5). We chose to explore these networks in depth to assess the context in which they were relevant to β-cell function in obesity.

The blue module is highly expressed in 20wk high-fat males, correlating with blood glucose concentration, and suggests enrichment for fatty acid metabolism genes (Fig. 5, **Supplemental Figure**S6, Table 3). In β-cells, fatty acid metabolism provides a glucose-independent stimulus for insulin release [61, 62]. While short term fatty acid exposure enhances GSIS, prolonged exposure decreases GSIS and reduces insulin content [61, 63]. This phenomenon is linked to the glucose-fatty acid cycle, where intense fatty acid oxidation inhibits glucose oxidation [64, 65]. Fatty acid metabolism promotes inflammation through low-density lipoprotein (LDL) and NF-κB activation, which drive angiogenesis [66–70]. One differentially connected gene in the blue network, *Snx25*, regulates inflammatory signaling through NF-κB-mediated transcription, providing a mechanism through which differences in *Snx25* activity (but not expression) result in differences in genetic network structure [71]. Expression of this network may be connected to the abundance of Beta 1 cells in 20k high-fat males, which are geared toward basal insulin release.

In summary, we identified 4 β-cell subpopulations whose relative proportions change depending on metabolic state. Beta 1 cells are primed for basal insulin secretion and proportionally high in hyperglycemic obese mice. Beta 2 cells are primed for protection from hypoxia associated with enhanced function and are abundant in normoglycemic obese and normoglycemic lean mice at the expense of Beta 1 cells. In conjunction, hyperglycemic obese mice express a highly connected genetic network associated with fatty acid metabolism, which is lost as glycemic control improves. The interplay between changing β-cell subpopulations and decreased fatty acid metabolism likely contributes to the improved β-cell function and subsequent restoration of glycemic control seen in obese SM/J mice [20, 21]. This study provides a road map for exploring cellular heterogeneity by integrating sc- and bulk RNA-seq data, allowing for robust characterization of subpopulation structure, differential expression, and network analysis associated with obesity and glycemic stress.

## Methods

### Metabolic phenotyping

SM/J mice were obtained from The Jackson Laboratory (Bar Harbor, ME). Mouse colony was maintained at the Washington University School of Medicine and all experiments were approved by the Institutional Animal Care and Use Committee in accordance with the National Institutes of Health guidelines for the care and use of laboratory animals. Mice were weaned onto a high-fat diet (42% kcal from fat; Envigo Teklad TD88137) or isocaloric low-fat diet (15% kcal from fat; Research Diets D12284), as previously described [21]. At 20 or 30 weeks of age, mice were fasted for 4 h, body weight measured, and blood glucose was measured via glucometer (GLUCOCARD). Mice were injected with sodium pentobarbital, followed by a firm toe pinch to ensure unconsciousness. Blood was collected via cardiac puncture and pancreas was collected. Blood was spun at 6000 rpm at 4 °C for 20 min to collect plasma. Insulin ELISA (ALPCO 80-INSMR-CH01) was used to measure plasma insulin levels following manufacturer’s instructions.

### Islet isolation and phenotyping

Islets were isolated from pancreas as previously described [20] and rested overnight. 5 Islets were equilibrated in KRBH buffer with 2.8 mM glucose for 30 min at 37 °C, then placed in 150 µl KRBH containing 2.8 mM glucose at 37 °C for 45 min, then 150 µl KRBH containing 11 mM glucose at 37 °C for 45 min. Islets were then transferred into 150 µl acid ethanol. Islet content and secretion tubes were stored at -20 °C overnight. Experiments were performed in duplicate per individual, and measurements are reported as the average of replicates. Mouse insulin ELISA (ALPCO 80-INSMU-E01) was performed, with the secretion tubes diluted 1:5, and content tubes diluted 1:100. Glucose stimulated insulin secretion was calculated by dividing insulin secretion at 11mM glucose by insulin secretion at 2.8 mM glucose. Basal insulin secretion was calculated by dividing insulin secretion at 2.8 mM glucose by islet insulin content. Total islet protein was measured using Pierce BCA Protein Assay kit (Thermo Scientific) according to manufacturer’s instructions and read at 562 nm on the Synergy H1 Microplate Reader (Biotek). Islet insulin content was calculated by dividing the islet insulin level in the content tubes by total islet protein. All measurements were taken in duplicate, values reported are the average of replicates.

### Single cell RNA sequencing

Single cell RNA sequencing (scRNA-seq) was performed on islets isolated from 15 SM/J mice representing 6 cohorts: 20wk high-fat females (n = 3), 20wk high-fat males (n = 3), 30wk high-fat females (n = 3), 30wk high-fat males (n = 2), 20wk low-fat females (n = 2), and 20wk low-fat males (n = 2). Isolated islets were dissociated into single cell suspensions using Accumax cell/tissue dissociation solution (Innovative Cell Technologies). Libraries were prepped using the Chromium Single Cell 3ʹ GEM, Library & Gel Bead Kit v3 (10xGenomics) and sequenced at 2 × 150 paired end reads using a NovaSeq S4. After sequencing, reads were de-multiplexed and assigned to individual samples. Reads were aligned using 10x Genomics CellRanger (3.1.0) against our custom SM/J reference [72]. Samples that were prepped together were aggregated into batches using CellRanger aggregate. In the R environment (4.0.0), each aggregated batch was run through SoupX (1.5.0) [73] to estimate and correct for ambient RNA contamination. A contamination fraction of 0.05 was chosen. Removal of *Ins2* ambient RNA shown in **Supplemental Figure**S1D-E. Adjusted counts were imported into Seurat (3.2.2) [74], where cells were filtered for number of features detected (500–3000), total counts detected (1000–30,000), percent mitochondrial genes (0–30), visualized in **Supplemental Figure**S1A. For additional quality control, we excluded cells where nCount was not predictive of nFeature; the predictive error (residual) of a cell had to be within 3 standard deviations of the mean predictive error (~ 0), resulting in 47,717 cells included in the analysis. Cell counts for samples from one batch shown before and after filtering step shown in **Supplemental Figure**S1B-C. Expression was then normalized in Seurat (normalization.method = LogNormalize), batches were integrated, and clustered using a shared nearest neighbor approach. Using the top 10 principal components of the filtered expression data and a resolution of 0.14 determined by the sc3 stability index, we identified 9 clusters of cells using Clustree (0.4.3) [75]. Cell types were assigned by identifying top over-expressed genes for each cluster relative to all other clusters using a Wilcoxon rank sum test, with an average log-fold-change threshold of > = 0.25 and requiring at least 25% of cells express the gene. Identities were assigned by comparing top over-expressed genes for each cluster with known cell-type specific markers for islet cells.

### Identification of β-cell subtypes, differential expression, and composition

All β-cells were subset for further analysis. Using the top 10 principal components of the filtered expression data and a resolution of 0.09 determined by the sc3 stability index (**Supplemental Figure**S1F), we identified 4 populations of β-cells, labeled Beta 1–4. Top over expressed genes for each population were identified using a Wilcoxon rank sum test, with an average log-fold-change threshold > = 0.1 compared to all other β-cells and an adjusted p-value < 0.01 (Bonferroni), shown in **Supplemental Table**S1. Subtype-identifier genes were tested for gene set enrichment (see below) to determine presumed function of each population. Using these thresholds (average log-fold-change > = 0.1 and adjusted p-value < 0.01), differential expression was calculated across all β-cells between 20wk high-fat males and 30wk high-fat males (**Supplemental Table**S2), between Beta 1 and Beta 2 subpopulations (**Supplemental Table**S3), and between Beta 1 cells across 20wk high-fat males and 30wk high-fat males (**Supplemental Table**S4) using the “MAST” hurdle-model test [76]. Relative proportions of β-cell subpopulations in each cohort was estimated using bootstrapping to calculate 95% confidence intervals by randomly sampling 1,000 cells from each cohort for 100 iterations.

### Identification of β-cell-specific genes

To identify β-cell-specific gene expression signatures in the bulk sequencing data, we assume that for a given gene, the sum of expression in the scRNA-seq data, *Y*_*Total*_, approximates the expression in bulk RNA sequencing data, *Y*_*Bulk*_.

$${Y}_{Bulk}\approx {Y}_{Total}$$

The *Y*_*Total*_ value can be re-written as the sum of expression from all the contributing cell types, where *Y* is the expression from a given cell type, and *N* is the total number of cells of that type.

$$\begin{array}{c}E\left[ {{Y_{Total}}} \right] \circ \sim \circ E\left[ {{Y_{\beta - cell}}} \right] * {N_{\beta - cell}} + E\left[ {{Y_{\alpha - cell}}} \right] * \\{N_{\alpha - cell}} + \, \ldots \, + \,E\left[ {{Y_{\beta - cell}}} \right] * {N_{\beta - cell}} + \end{array}$$

Therefore, the expected relative contribution of β-cells (*Q*_*β−cell*_), to total expression (*Y*_*Total*_), can be written as:

$${Q_{B - Cell}} \circ \sim \circ \frac{{E\left[ {{Y_{\beta - cell}}} \right] * {N_{\beta - cell}}}}{{E\left[ {{Y_{Total}}} \right]}}$$

When *Q*_*β−cell*_ is high in all 6 cohorts, we are confident that gene expression in the bulk data is nearly exclusively coming from β-cells. To determine the contribution of β-cells and all other cell types to gene expression, the distribution of normalized expression was assessed using a Cullen and Frey graph using the fitditrplus package (1.1-3) [77] in R and identified as beta distributed (**Supplemental Figure**S2A-B).

scRNA-seq data is characterized by a buildup of expression values at 0 due to cells that do not express a gene or due to gene-dropout. This distribution required us to employ a beta-hurdle model described by three parameters: Pr(Dropout), α, and β. We treat the probability of attaining an expression value of 0 ( Pr(Dropout) ) and the distribution of non-zero values separately for a given gene. We fit the expression of each gene in each cell type using a beta-hurdle model optimized by maximum goodness of fit estimation. To fit the first part of the beta-hurdle mode, the Kolmogorov-Smirnov statistic using the ks.test package (4.05) [78] in R was used to select the optimal α and β shape parameters that best fit the expression distribution of non-zero-expressing cells by minimizing the distance between the cumulative distribution of the real data and theoretical beta model data (Ks) (**Supplemental Figure**S2C). To fit the second part of the beta-hurdle mode, Pr(Dropout) was iterated between 0 and 1, selecting the Pr(Dropout) that minimized Ks (**Supplemental Figure**S2D). In most cases, assuming 100% of cells not expressing the gene was due to gene drop out provided the best fit (**Supplemental Figure**S2E). From the best fit beta-hurdle model, the expected value for each gene within a cell type $E\left[{Y}_{CellType}\right]$ can be calculated using the fit α and β shape parameters as:

$$E\left[{Y}_{CellType}\right]=\frac{1}{1+\left(\frac{\beta }{\alpha }\right)}$$

Multiplying the expected value by the total number of cells of that cell type provides the total expression contribution of that cell type. Summing this value across all cell types provides total expression and allows assessment of the contribution of β-cell gene expression to the genes’ total expression. We required β-cells to account for > = 80% of total gene expression in each of the six cohorts analyzed, resulting in 316 “β-cell-specific genes”, shown in **Supplemental Table**S5.

### Bulk RNA sequencing

Islets from 32 mice were sequenced: 4 males and 4 females from each diet (high-fat and low-fat) and each age (20wk and 30wk), n = 32. Islet RNA was extracted using the RNeasy MinElute Cleanup kit (Qiagen), RNA concentration was measured via Nanodrop and RNA quality/integrity was assessed with a BioAnalyzer (Agilent). Libraries were prepped using the SMARTer cDNA synthesis kit (Takara Bio) and sequenced at 2 × 150 paired end reads using a NovaSeq S4. After sequencing, reads were de-multiplexed and assigned to individual samples. FASTQ files were trimmed and filtered to remove low quality reads and aligned against a SM/J custom genome using STAR [72, 79, 80]. Read counts for β-cell-specific genes were normalized via TMM normalization and pairwise differential expression between cohorts was performed using edgeR [81]. Differential expression analysis for all 316 β-cell-specific genes across select cohort comparisons reported in **Supplemental Table**S6.

### Co-expression network analysis

Weighted Gene Co-Expression Network Analysis (WGCNA) identifies co-expression modules and correlates them with phenotypic traits [22]. Briefly, edgeR-normalized counts for β-cell-specific genes were converted to standard normal, and an adjacency matrix was created from bi-weight mid-correlations calculated between all genes in all individuals and raised to a power β of 8, chosen based on a scale-free topology index above 0.9, to emphasize high correlations [82]. The blockwiseModules function created an unsigned Topological Overlap Measure using the adjacency matrix to identify modules of highly interconnected genes. Eigengenes were calculated as the first principal component for each module, and Pearson’s correlations were calculated between eigengene expression and phenotype to estimate module-trait relationships. Module-trait correlations were considered significant at an FDR-corrected p-value < 0.05. Each gene’s module membership and correlation with metabolic phenotypes are reported in **Supplemental Table**S7. The adjacency matrix was then used to calculate the connectivity of each gene with other genes within its module (k_within_). Adjacency matrices were subset for each age x diet x sex cohort, used to calculate cohort-specific connectivity for each gene (**Supplemental Table**S8), then used to calculate differential connectivity between cohorts. We report differential connectivity between 20wk high-fat males and 30wk high-fat males, *HM*, calculated as

$$kDiff\left(HM\right)= \frac{HM20-HM30}{HM20+HM30}$$

where HM20 and HM30 are cohort-specific k_within_ values for each gene. This provides each gene in the comparison with a value between − 1 and 1, where |kDiff| > 0.5 is considered differentially connected. Genes with positive differential connectivity are more highly connected in 20wk high-fat males than 30wk high-fat males.

### Gene set enrichment analysis

Over-representation analysis (ORA) was performed using the WEB-based Gene Set Analysis Toolkit v2019 [83] on genes overexpressed in individual β-cell subtypes, β-cell-specific genes, and genes within the blue expression module, using all genes expressed in the sc-RNAseq data set as a reference set. Analysis included gene ontologies (biological process, cellular component, molecular function), pathway (KEGG), and phenotype (Mammalian Phenotype Ontology).

## Electronic supplementary material

Below is the link to the electronic supplementary material.

Supplemental Figures 1–6

Supplemental Table 1

Supplemental Table 2

Supplemental Table 3

Supplemental Table 4

Supplemental Table 5

Supplemental Table 6

Supplemental Table 7

Supplemental Table 8

## Data Availability

All sequencing data is available through the National Library of Medicine Sequencing Read Archive (SRA), accession number: PRJNA751057. All results are available as Supplementary Tables.
