# Supplementary material for: Integrated transcriptomics contrasts fatty acid metabolism with hypoxia response in β-cell subpopulations associated with glycemic control
Source: BMC Genomics. 2023 Mar 28;24:156. doi: 10.1186/s12864-023-09232-5 (PMC10052828; doi:10.1186/s12864-023-09232-5)
Supplement: Supplementary file 1 — Supplemental Figures 1–6 [file 12864_2023_9232_MOESM1_ESM.docx]

Table of Contents

**Supplemental Figure 12**

**Supplemental Figure 23**

**Supplemental Figure 34**

**Supplemental Figure 45**

**Supplemental Figure 56**

**Supplemental Figure 67**


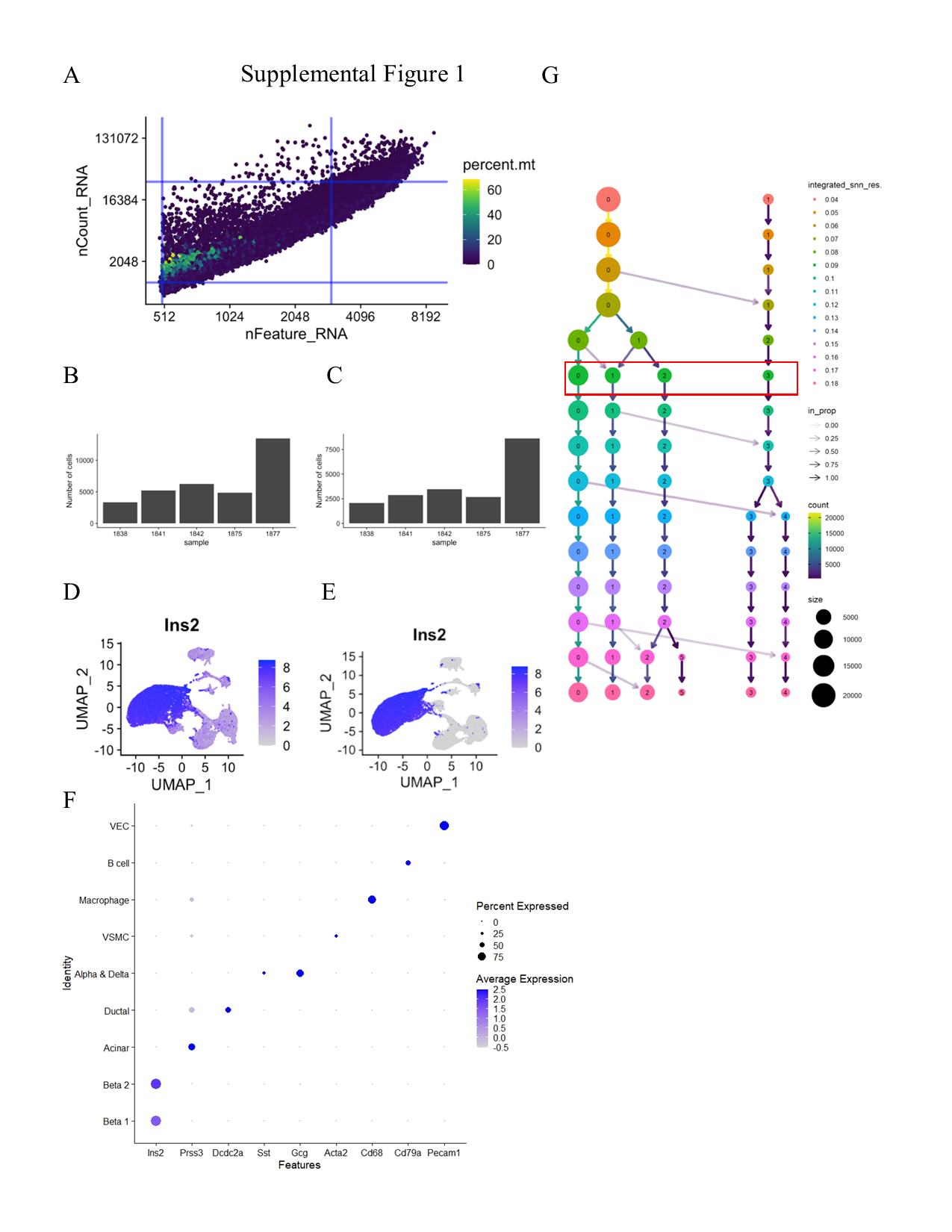


**Supplemental Figure 1**. Single cell RNA sequencing quality control. **(A)** Total counts, features, and mitochondrial RNA expression plotted for each cell. Cells within blue box and without mitochondrial gene expression were included in analysis. **(B)** Number of cells identified in individual mice prior to quality control, **(C)** and after quality control. **(D)** Expression of *Ins2* across all cells prior to ambient RNA removing using SoupX, **(E)** and after ambient RNA removal. **(F)** Dot plot depicting expression of cellular marker genes used to determine cluster identity. **(G)** Resolution analysis to determine the number of β-cell subpopulations. Selected resolution highlighted with red box.


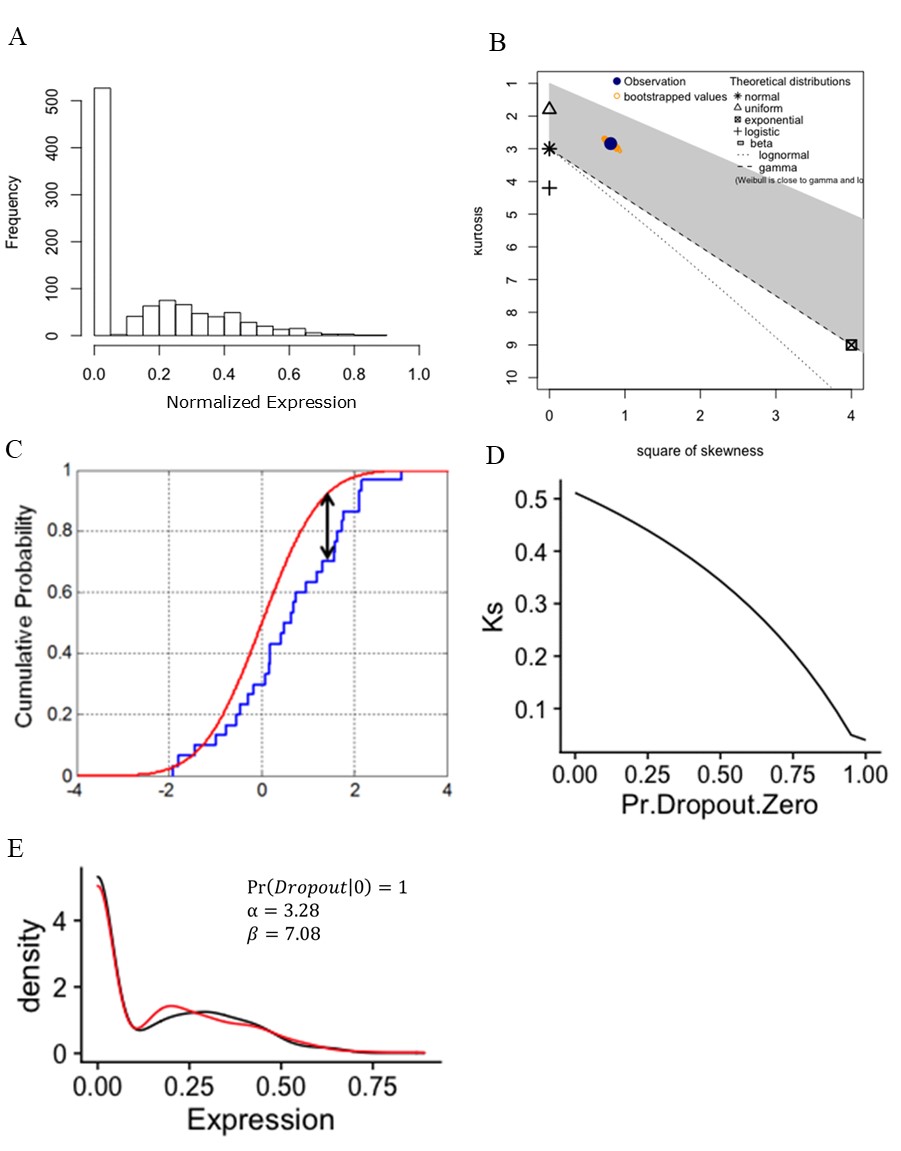


**Supplemental Figure 2.** Quantifying expected expression for β-cell expression of *Dcd*. **(A)** Distribution of expression across all β-cells. **(B)**  Cullen and Frey analysis identifies *Dcd* expression to be beta distributed. **(C)** Kolmogorov-Smirnov test identifies alpha and beta parameters that minimize Ks between real (blue line) and simulated (red line) distribution of cells expressing *Dcd*. **(D)** Estimating percent of cells not expressing *Dcd* due to gene drop out by iterating alpha and beta parameters that minimize Ks between real and simulated data. **(E)** Density plot visualizing estimated parameters for distribution of *Dcd* expression. Red line shows distribution of actual data, black line shows distribution of simulated data based on optimal alpha and beta parameters. From these parameters, expected expression is calculated.

**
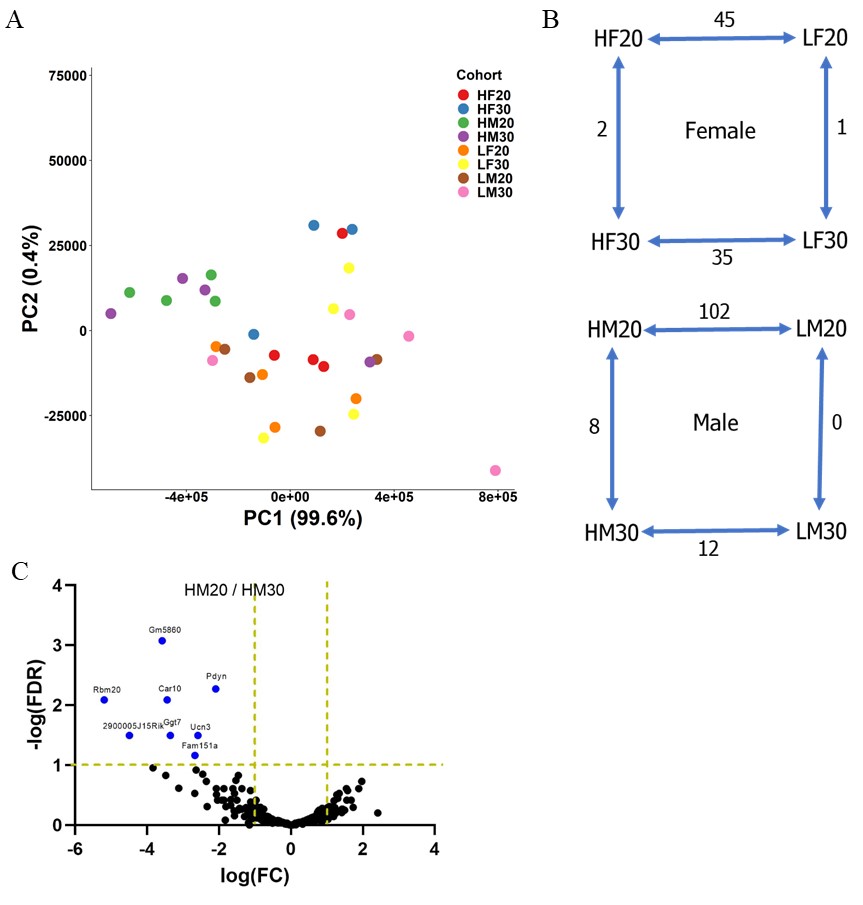
**

**Supplemental Figure 3.** β-cell-specific gene *Pdyn* is differentially expressed in obese males. **(A)** Principal component analysis of bulk RNA sequencing data normalized with only β-cell-specific genes. **(B)** Number of differentially expressed genes in females and males, across diets and age. **(C)** Differentially expressed genes between 20wk high-fat males and 30wk high-fat males. Vertical golden lines indicate threshold for significance based on average log fold change, horizontal line indicates threshold for significance based on FDR corrected p-value. . Blue genes are significantly under-expressed in comparison. HF20 – 20wk high-fat female, HF30 – 30wk high-fat female, HM20 – 20wk high-fat male, HM30 – 30wk high-fat male, LF20 – 20wk low-fat female, LM20 – 20wk low-fat male, LF30 – 30wk low-fat female, LM30 – 30wk low-fat male.


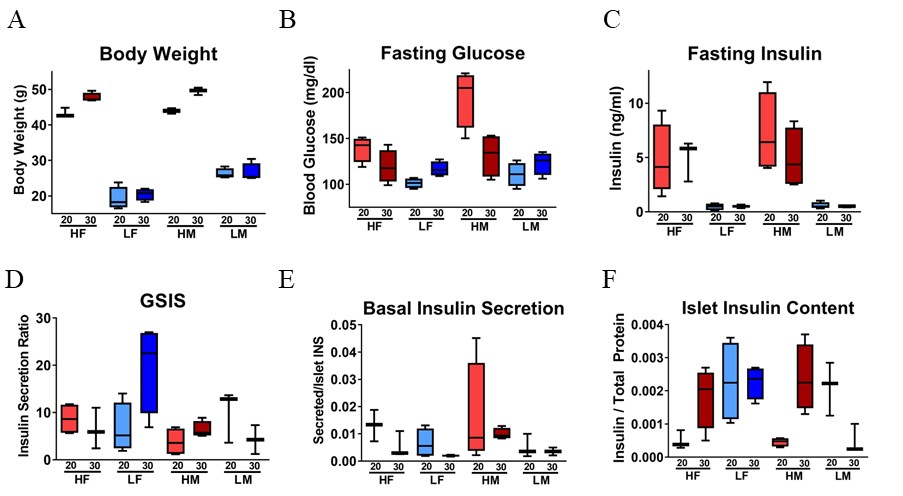


**Supplemental Figure 4**. Metabolic and islet phenotypes from bulk RNA sequencing mice. **(A)** body weight, **(B)** blood glucose, **(C)** serum insulin levels collected after 4-hour fast. **(D)** Glucose-stimulated insulin secretion (GSIS), **(E)** Basal insulin secretion, **(F)** Islet insulin content collected after isolated islets were rested overnight. N = 4 mice per age X sex X diet cohort. N= 10 islets per individual for islet phenotypes. Middle bar represents mean, box represents 25^th^ and 75^th^ quartile, whiskers represent minimum and maximum values. HF20 – 20wk high-fat female, HF30 – 30wk high-fat female, HM20 – 20wk high-fat male, HM30 – 30wk high-fat male, LF20 – 20wk low-fat female, LM20 – 20wk low-fat male, LF30 – 30wk low-fat female, LM30 – 30wk low-fat male.


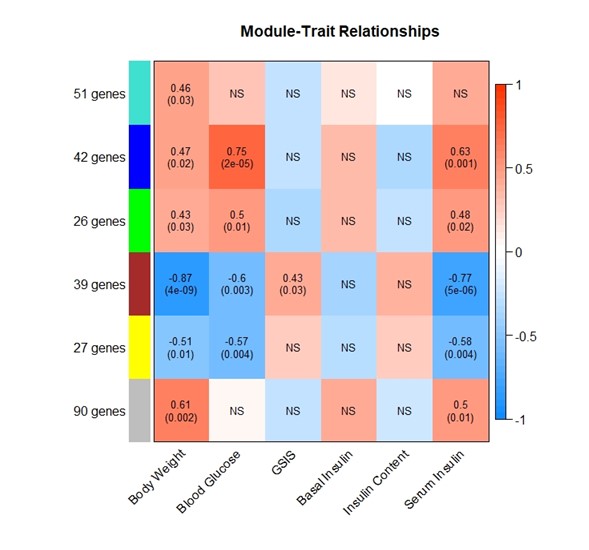


**Supplemental Figure 5.** Correlation between phenotype and module eigengene expression. Number of genes within each module reported on y-axis, phenotypic trait reported on x-axis. For each module-trait relationship, the Pearson correlation between eigengene expression and phenotype value is reported (top) along with an FDR-corrected p-value for the correlation (bottom). Color of box indicates strength of correlation. NS - non-significant association based on FDR-corrected p-value.


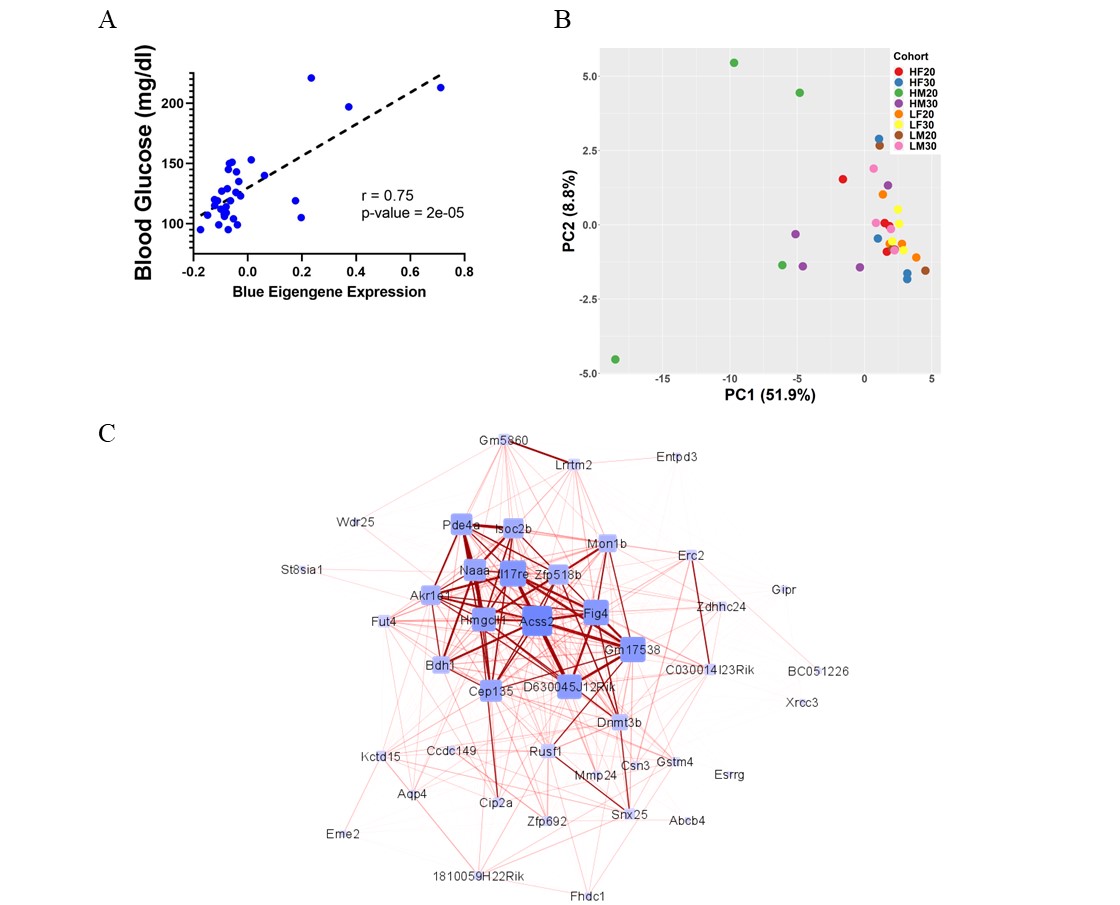


**Supplemental Figure 6**. Overview of blue module. (**A**) Correlation between blue module eigengene expression and blood glucose levels across all cohorts. (**B**) Principal component analysis of gene expression within the blue module, segregated by cohort. (**C**) Blue module network structure in across all cohorts. Size and color of node indicates overall connectivity within the network, thickness of edges indicates strength of correlation between gene pairs.
